# Moderate iron deficiency and high dietary iron intake differentially alter hepatic lipid metabolism and adipose tissue lipid handling in mice

**DOI:** 10.3389/fnut.2025.1725052

**Published:** 2026-01-27

**Authors:** Shangjie Wu, Pengwei Li, Qian Liu, Keying Zhang, Xuemei Ding, Jianping Wang, Qiufeng Zeng, Yan Liu, Yue Xuan, Shanshan Li, Yadong Mu, Shiping Bai

**Affiliations:** Key Laboratory of Animal Disease-Resistance Nutrition, Ministry of Education, Ministry of Agriculture and Rural Affairs, Key Laboratory of Sichuan Province, Animal Nutrition Institute, Sichuan Agricultural University, Chengdu, China

**Keywords:** adipose tissue, *de novo* lipogenesis, hepatic steatosis, iron homeostasis, lipid metabolism, mice, mitochondrial dysfunction

## Abstract

**Background:**

Iron (Fe) is an essential micronutrient, yet both its deficiency and overload have been associated with disruptions in lipid metabolism. This study investigated the effects of moderate iron deficiency and high dietary iron on lipid metabolic pathways in mice.

**Methods:**

Five-week male C57BL/6J mice were fed for 16 weeks on one of three diets: a basal iron-deficient diet without iron supplementation (FeD, 19.26 mg/kg Fe), and the same basal diet supplemented with either 200 mg Fe/kg (iron-adequate control, Control) or 1,200 mg Fe/kg (high-iron, FeH). Growth performance, iron status, serum lipids, tissue iron deposition, hepatic fatty acid composition, and expression of key genes and enzymes involved in lipid metabolism were analyzed.

**Results:**

The FeD group exhibited increased body weight and feed intake, and reduced systemic iron parameters. Molecular analysis revealed a distinct pattern of lipid metabolic disruption in FeD, characterized by the upregulation of certain hepatic lipogenic transcripts (*ACLY, SREBP1c, PPAR*γ) but without a concomitant increase in functional lipogenic output or hepatic triglycerides. Notably, the elevation in SCD1 protein occurred alongside a decreased hepatic C18:1 n-9/C18:0 ratio in the FeD group. In adipose tissue, FeD specifically enhanced lipolysis gene expression (*ATGL, HSL, FABP4*), indicating elevated lipid mobilization. In contrast, FeH mice developed hyperlipidemia and hepatic iron overload, which was driven by direct activation of the hepatic *SREBP1c* pathway and its lipogenic targets (*ACC, FAS, SCD1*). *Hamp* expression was significantly upregulated in the FeH group compared to both the control and FeD groups (*p* < 0.05). Although both diets altered hepatic fatty acid composition, they operated through fundamentally distinct mechanisms.

**Conclusions:**

These findings demonstrate that moderate iron deficiency and high iron intake disrupt hepatic lipid metabolism via different pathways: FeD primarily through systemic adaptations leading to post-translational constraints on iron-dependent enzymes, whereas FeH acts through direct transcriptional activation of hepatic *de novo* lipogenesis, potentially involving hepcidin-mediated cross-talk. The study underscores the critical importance of iron homeostasis in preventing dyslipidemia and hepatic steatosis and provides mechanistic insights that could inform dietary recommendations for populations at risk of metabolic disorders.

## 1 Introduction

Iron (Fe) is an essential trace element that plays a fundamental role in mammalian energy metabolism and numerous enzymatic processes (1). Disruptions in iron homeostasis, encompassing both deficiency and overload, are increasingly recognized as modulators of systemic metabolism and contributors to diseases such as non-alcoholic fatty liver disease (NAFLD). Fe deficiency remains one of the most prevalent nutritional disorders worldwide, affecting over 2 billion people and posing significant public health challenges (2). Clinical and epidemiological studies reveal a complex relationship between iron status and NAFLD. Iron deficiency is common in obesity, potentially influencing energy metabolism and lipid handling, while mild to moderate hepatic iron overload, found in a subset of NAFLD patients, is associated with more severe steatosis and fibrosis. This bidirectional relationship suggests that iron acts as a metabolic modulator, impacting pathways relevant to lipid synthesis, storage, and oxidation. Clinically, Fe deficiency has been correlated with alterations in lipid profiles and essential fatty acid metabolism, though mechanistic understanding remains incomplete (3). Experimental studies in rodents have demonstrated that severe Fe deficiency can profoundly disrupt lipid homeostasis (4–6). Conversely, Fe supplementation, commonly used to prevent deficiency, has been linked to excess Fe accumulation, which may promote hepatic steatosis and dyslipidemia (7). Severe Fe overload is known to induce oxidative stress, lipid peroxidation, and impaired fatty acid desaturation (8, 9). However, it remains unclear whether iron deficiency and excess converge on hepatic lipid accumulation via similar or fundamentally distinct pathways.

In typical human diets, Fe content varies substantially; cereal grains provide 30–140 mg Fe/kg dry matter, while cereal by-products may contain 220–2,600 mg Fe/kg (10). Although the recommended daily Fe intake is 12–18 mg for most age groups in North America (11), moderate Fe deficiency is far more common in clinical settings than the severe deficiency often reproduced in rodent models. While the metabolic impacts of severe iron deficiency have been documented (4–6), the consequences of moderate iron deficiency—a condition far more clinically prevalent—on a comprehensive set of lipid metabolic parameters remain less defined and are often conflated with those of severe deficiency.

The liver serves as a central organ for both Fe storage and lipid metabolism. Fe status has been shown to modulate hepatic lipid storage, secretion, and metabolic flux (5, 7, 12). While Fe deficiency has been associated with enhanced lipogenesis and Fe overload with oxidative damage and NAFLD progression, the comparative impact of moderate deficiency vs. high intake on key molecular drivers remains poorly defined. To address this gap and mechanistically dissect the distinct pathogenic pathways, we strategically selected a panel of biomarkers spanning the lipid regulatory network: fatty acid binding protein (FABP) 1 for fatty acid uptake, sterol regulatory element-binding protein 1c (SREBP1c) and peroxisome proliferator-activated receptor-γ (PPARγ) as the master transcriptional regulators of lipogenesis, their key enzymatic targets acetyl-CoA carboxylase (ACC) and fatty acid synthase (FAS) for *de novo* lipogenesis flux, and the Fe-dependent enzymes stearoyl-CoA desaturase 1 (SCD1) and aconitase (ACO) to directly assess the functional consequences of altered Fe availability on fatty acid desaturation and mitochondrial metabolism, respectively (13–18). We hypothesize that FeD and FeH disrupt hepatic and adipose tissue lipid metabolism through fundamentally different pathways. While this study focuses on male mice to control for the confounding effects of the estrous cycle, future investigations in females will be crucial for a complete understanding of iron-lipid interplay.

## 2 Materials and methods

### 2.1 Animals, diets, and experimental design

All experimental protocols were approved by the Animal Care Committee of Sichuan Agricultural University (Protocol No. SAU20220012). Only male C57BL/6J mice were procured from Chengdu Dossy Experimental Animals Co., Ltd. (Chengdu, China) and used in this study. This decision was made to minimize metabolic variability associated with the female estrous cycle, as estrogen fluctuations are known to significantly influence both iron and lipid homeostasis (19, 20). Before the experiment, mice were acclimatized with free access to a standard commercial diet (SCD1005; Fe content: 226.5 mg/kg) and tap water (0.06 mg/L).

At 5 weeks of age (mean body weight 18.28 ± 1.59 g), thirty male mice were randomly divided into three dietary groups. The three treatments included a basal Fe-deficient diet (FeD; 19.26 mg Fe/kg by analysis), a control diet supplemented with 200 mg Fe/kg [simulating typical Fe content in standard rodent diets (21)], and a high-Fe diet supplemented with 1200 mg Fe/kg (FeH). The selected FeH dose (about 5–6 times the standard rodent diet) models a scenario of high dietary iron intake, relevant to populations with high consumption of iron-fortified foods or supplements, which has been epidemiologically linked to adverse metabolic outcomes (22). The FeD level was set to induce moderate deficiency without anemia, mirroring a prevalent subclinical condition often undetected in clinical settings. Each group contained 10 mice and each one was as one experimental unit. The mice were fed different diets for 16 weeks. The basal diet was formulated according to AIN-93G guidelines (23), excluding Fe, and comprised corn starch, casein, maltodextrin, sucrose, soybean oil, *L*-cystine, cellulose, vitamin and mineral premixes (Fe-free), and sodium chloride (see Supplementary Table 1). Macronutrient composition was as follows: 18.30% crude protein, 7.10% crude fat, 5.00% crude fiber, 63.20% carbohydrate, 0.50% calcium, and 0.20% phosphorus. Ferric ammonium citrate (99.99% purity) was added to achieve target Fe concentrations.

### 2.2 Animal housing and husbandry

The mice were singly housed in plastic-coated stainless steel cages (365 × 207 × 140 mm) following the experimental procedures to prevent aggression and ensure accurate monitoring of individual food intake and physiological responses. The animal facility maintained a 12/12-h light/dark cycle (lights on at 7:00 AM), a temperature of 22 °C ± 2 °C, and relative humidity of 50% ± 10%. All mice were provided with *ad libitum* access to the different experimental diets and deionized water. Aspen wood chip bedding was used and changed twice a week. To promote welfare and minimize stress induced by single housing, enhanced environmental enrichment was consistently provided in each cage, including ample nesting material, a red transparent plastic shelter, and a wooden chew block. Mice were acclimatized to these housing conditions for at least 7 days prior to any experimental procedures.

### 2.3 Growth performance

Body weight gain (BWG) and total feed intake (FI) were calculated for the entire 16-week experimental period. The feed conversion ratio (FCR) was calculated for the entire period using the formula: FCR = total FI/BWG.

### 2.4 Sample collection

Animals were monitored daily for general health and well-being. After a 16-week feeding period, all mice were fasted for 12 h, weighed, and then euthanized for sample collection. The mice were euthanized by exposure to a rising concentration of carbon dioxide (CO_2_) in a transparent induction chamber. CO_2_ was delivered from a compressed gas cylinder using a calibrated flowmeter to achieve a fill rate of 30% of the chamber volume per minute. Following respiratory arrest, death was confirmed by cervical dislocation, in accordance with the American Veterinary Medical Association (AVMA) Guidelines. After euthanasia, the blood was collected from all mice via cardiac puncture. Approximately 0.2 ml was placed in an EDTA-coated tube for hematological analysis. The remaining blood was clotted at room temperature for 40 min, centrifuged at 3,000 rpm for 10 min, and the serum was stored at −80 °C. After blood collection, the liver, epididymal, perirenal, and retroperitoneal adipose tissues were dissected, weighed, and immediately snap-frozen in liquid nitrogen to preserve RNA and protein integrity. All snap-frozen samples were then stored at −80 °C until further analysis. Tissue weights are expressed as a percentage of final BW.

### 2.5 Blood biochemical parameters

Hematocrit and hemoglobin levels were measured using an automated hematology analyzer (DxH 500; Beckman Coulter, Inc., Brea, CA, USA). Serum Fe and unsaturated Fe-binding capacity (UIBC) were determined colorimetrically using an automatic biochemical analyzer (Hitachi 3000; Hitachi High-Technologies Corporation, Tokyo, Japan) with their respective commercial kits. Total Fe-binding capacity (TIBC) was calculated as serum Fe + UIBC, and transferrin saturation (TS) as (serum Fe/TIBC) × 100%. Serum ferritin was quantified using a species-specific enzyme-linked immunosorbent assay (ELISA) kit (Bioleaf Biotech, Shanghai, China). Lipid parameters including total cholesterol (TC), triglycerides (TAG), high-density lipoprotein cholesterol (HDLC), and low-density lipoprotein cholesterol (LDLC) were analyzed enzymatically using the same biochemical analyzer with corresponding commercial kits (Nanjing Jiancheng Bioengineering Institute, China). Very low-density lipoprotein cholesterol (VLDL) was estimated as TC—(HDLC + LDLC), a widely used calculated estimate in rodent metabolic studies (24). While this method provides a functional assessment, direct quantification would offer higher precision. Serum non-esterified fatty acids (NEFA) were measured with an ELISA kit (Cell Biolabs, Inc., Beijing, China) as previously described (25). Detailed specifications of all commercial kits including catalog numbers, assay principles, and manufacturer's validation data for mouse samples are provided in Supplementary Table 2.

### 2.6 Fe concentration in tissues

The Fe content in the diet and tissue samples (liver, spleen, kidney, and heart) was determined by flame atomic absorption spectroscopy (FAAS) using an atomic absorption spectrometer (model AA700, Jana Analytical Instrument Co., Ltd., Beijing, China). Approximately 0.2 g of tissue or 0.5 g of diet sample was accurately weighed and digested high-purity nitric acid in a closed-vessel microwave digestion system (MARS 6, CEM Corporation, Matthews, NC, USA). A two-step heating protocol was applied: the temperature was first raised to 120 °C and held for 10 min, then further increased to 180 °C for a 20-min hold. The resulting digests were then evaporated to near-dryness under a gentle nitrogen stream. The residues were quantitatively transferred and diluted with 0.5% (v/v) nitric acid to a final volume for analysis. Method accuracy was validated by digesting and analyzing a certified bovine liver reference material (National Institute of Standards, Beijing, China), which was treated identically to the samples.

### 2.7 Hepatic lipid and fatty acid analysis

Hepatic TC and TAG concentrations were quantified using enzymatic colorimetric kits. Their concentration values were normalized to total protein concentration rather than tissue wet weight. This approach controls for potential diet-induced differences in hepatic composition (e.g., lipid or water content), thereby more accurately reflecting triglyceride accumulation per unit of metabolically active tissue. For fatty acid composition analysis, total lipids were extracted from approximately 100 mg of liver tissue using a modified protocol. To prevent oxidation, 0.01% (w/v) butylated hydroxytoluene (BHT) was added to all organic solvents. The internal standard, heptadecanoic acid (C17:0, 50 μg), was added to the tissue prior to homogenization to correct for extraction efficiency. The tissue was homogenized in 2 ml of a chloroform-methanol mixture (2:1, v/v) using a tissue homogenizer. After vortexing for 10 min and subsequent centrifugation at 3,000 × g for 10 min, the organic (lower) phase was carefully transferred to a new tube. The extraction was repeated once with an additional 2 ml of the chloroform-methanol mixture. The combined organic phases were evaporated to dryness under a stream of nitrogen gas.

The extracted lipids were derivatized to fatty acid methyl esters (FAMEs) via a two-step reaction. First, saponification and methylation were initiated by adding 1 mL of 0.5 M KOH in methanol and incubating at 70 °C for 20 min. Subsequently, esterification was completed by adding 2 mL of 14% boron trifluoride (BF3) in methanol and further incubating at 70 °C for 30 min. After cooling, the FAMEs were extracted twice with 2 mL of hexane. The combined hexane extracts were evaporated to dryness under a gentle stream of nitrogen gas and reconstituted in 200 μl of hexane for GC-MS analysis.

FAME analysis was performed using a gas chromatography-mass spectrometry system (GC-MS; Agilent 7000E, Santa Clara, CA, USA) equipped with a DB-23 capillary column (60 m × 0.25 mm i.d., 0.25 μm film thickness). High-purity helium was used as the carrier gas at a constant flow rate of 1.0 ml/min. The injector temperature was set at 250 °C, and 1 μl of sample was injected in split mode (split ratio 10:1). The oven temperature program was as follows: initial temperature 100 °C held for 2 min, ramped to 240 °C at 4 °C/min, and held at 240 °C for 15 min. The mass spectrometer was operated in electron ionization (EI) mode at 70 eV. Fatty acids were identified by comparing their retention times and mass spectra with those of authentic standards, and quantified relative to the internal standard (C17:0) peak area. Calibration curves were constructed using unsaturated and saturated fatty acid standards (0.25–125 μg/ml and 0.1–100 μg/ml, respectively), with heptadecanoic acid (C17:0) as an internal standard.

### 2.8 Enzymatic activities and protein expression

Rationale for Assay Selection: the measurement approach (activity vs. concentration) was selected based on the biological question and analyte properties. The activities of metabolic enzymes FAS, ACC, and glycerol-3-phosphate dehydrogenase (G3PDH) were assessed to reflect the functional flux through the lipogenic pathway. In contrast, the protein levels of citrate synthase (CS), ACO, and SCD1 were quantified as established proxies for enzyme abundance and mitochondrial content.

Tissue Homogenization: liver tissue (0.1 g) was homogenized on ice in 1 mL of ice-cold lysis buffer (RIPA buffer supplemented with protease and phosphatase inhibitors) using a mechanical homogenizer (Tissuelyser LT, Qiagen) at 30 Hz for 2 min. The homogenate was then centrifuged at 12,000 × g for 30 min at 4 °C, and the supernatant (cytosolic fraction) was collected for subsequent assays.

Enzymatic Activity and Protein Assays: all measurements were performed using commercial kits according to the manufacturers' instructions. Detailed specifications of all commercial kits including catalog numbers, assay principles, and manufacturer's validation data for mouse samples are provided in Supplementary Table 3. Enzyme activities were defined as follows: FAS activity was measured via NADPH consumption and is expressed as nmol NADPH consumed per min per mg protein; G3PDH activity was measured via NAD^+^ reduction and is expressed as nmol NADH generated per min per mg protein; ACC activity was quantified using a coupled enzyme assay that links malonyl-CoA production to NADH oxidation, and is therefore expressed as nmol NADH consumed per min per mg protein. The protein concentrations of CS, ACO, and SCD1 were quantified using sandwich ELISA kits (for details, see Supplementary Table 3). Briefly, all kits employ matched antibody pairs for target capture and detection using HRP-conjugated antibodies, with colorimetric development by TMB substrate. All kits were validated by the manufacturers for use with mouse liver tissue samples. Total protein concentration in the supernatant was determined with Bradford reagent (Sigma-Aldrich, USA) for normalization. All measurements were performed in duplicate.

### 2.9 Quantitative real-time PCR

Total RNA was extracted from liver and epididymal adipose tissue using TRIzol reagent (Takara, China). RNA concentration and purity were assessed using a NanoDrop spectrophotometer (Thermo Fisher Scientific, USA). All samples had an A260/A280 ratio between 1.8 and 2.1 and an A260/A230 ratio >2.0, indicating high-purity RNA without significant protein or organic solvent contamination. RNA integrity was confirmed by 1.5% agarose gel electrophoresis, displaying clear 18S and 28S rRNA bands. To eliminate genomic DNA contamination, 1 μg of total RNA was treated with DNase I (RNase-free, Thermo Fisher Scientific) prior to cDNA synthesis.

cDNA was synthesized from 1 μg of DNase-treated RNA using the PrimeScript™ RT reagent kit (Catalog # RR037Q, TaKaRa, China), which contains oligo(dT) and random hexamer primers. The reverse transcription conditions were revised according to the manufacturer's protocol: 25 °C for 3 min (priming), 50 °C for 15 min (reverse transcription), followed by 85 °C for 5 sec (enzyme inactivation).

Real-time PCR was performed using SYBR Green Master Mix (Catalog # QP-01012, Foregene, < city>Chengdu < /city>, China) on a QuantStudio 5 Real-Time PCR System (Applied Biosystems). The genes selected for mRNA expression analysis represent key regulators and enzymes in pathways previously linked to iron metabolism, including lipogenesis (*SREBP1c, PPAR*γ, ATP-citrate lyase (*ACLY*)*, ACC, FAS, SCD1*), fatty acid uptake and mobilization (*FABP1*, cluster of differentiation 36 (*CD36*), lipoprotein lipase (*LPL*), adipose triglyceride lipase (*ATGL*), hormone-sensitive lipase (*HSL*)), mitochondrial function (*ACO, CS*), and lipid export/oxidation (apolipoprotein B (*ApoB*) and carnitine palmitoyl acyl-CoA transferase (*CPT1*)). This targeted approach was designed to test specific hypotheses regarding iron-lipid crosstalk while maintaining statistical power and interpretability. Gene-specific primers (sequences listed in Supplementary Table 4) were designed using NCBI Primer-BLAST and spanning exon-exon junctions where possible to further preclude genomic DNA amplification. The amplification efficiency for each primer pair was validated by generating a standard curve from a 10-fold serial dilution of cDNA, and only primers with efficiencies between 90% and 110% (with an *R*^2^ > 0.99) were used. Amplification conditions were: 95 °C for 30 s, followed by 40 cycles of 95 °C for 5 s and 60 °C for 34 s. The stability of the reference gene, β-actin, was validated using the NormFinder algorithm, and it exhibited stable expression across all experimental groups (M-value < 0.5). The 2^−ΔΔCt^ method was used to calculate relative gene expression levels, normalized to β-actin. All reactions were run in triplicate.

### 2.10 Statistical analysis

The sample size (*n* = 10 per group) was chosen based on the effect sizes observed in systemic iron status indicators (serum iron and ferritin) from our previous data (26) and is consistent with sample sizes used in similar nutritional studies in mice (15, 27). A *post hoc* power analysis performed with G^*^Power software (version 3.1.9.7) confirmed that this sample size provided >80% statistical power (α = 0.05) to detect the significant differences in key outcome, including plasma and hepatic TAG levels, along with pivotal hepatic enzymes and genes regulating lipid metabolism.

Statistical analyses were performed using SAS software (version 9.2). All data are expressed as mean ± standard deviation (SD). All datasets were first assessed for normality of distribution using the Shapiro-Wilk test and by visual inspection of Q–Q plots and histograms. And then, the homogeneity of variances was assessed using Levene's test. For the data that satisfied both assumptions, one-way analysis of variance (ANOVA) was employed followed by Tukey's honestly significant difference (HSD) *post hoc* test for multiple comparisons. Where the assumptions for parametric testing were violated, the non-parametric Kruskal–Wallis tests were applied, with subsequent pairwise comparisons performed using Dunn's test. To control for multiple comparisons across the large number of variables examined, the Benjamini-Hochberg false discovery rate (FDR) procedure was applied where appropriate, and key findings remained significant at an FDR of 5% (*q* < 0.05). A probability level of *p* < 0.05 was considered statistically significant. Sample randomization was employed for group assignment. For endpoint analyses where feasible (e.g., qPCR data analysis, enzymatic activity calculations, FAAS and GC-MS data processing), samples were coded, and the analysts were blinded to the group identity.

## 3 Results

### 3.1 Growth performance and adipose tissue depot weights

Compared with the control group, final BW was not significantly altered by FeH treatment. In contrast, FeD significantly increased BW after the 16-week feeding period (*p* < 0.05; Table 1). Throughout the experimental period (weeks 5–20), FeD resulted in significantly higher feed intake compared with both the control and FeH groups (*p* < 0.05), while FeH showed no notable effect. The BWG was significantly elevated in the FeD group relative to the control, accompanied by a reduced feed intake-to-BWG ratio (FI:BWG; *p* < 0.05). A similar tendency was observed in FeH-treated mice (*p* < 0.05), which exhibited a trend toward increased BWG and decreased FI:BWG. Both FeD and FeH significantly increased the absolute and relative (to BW) weights of epididymal and perirenal adipose depots (*p* < 0.05; Table 1). In contrast, neither dietary Fe level had a significant effect on retroperitoneal fat mass.

**Table 1:** Dietary iron intake influences growth performance and adipose tissue deposition in male mice.

**Items^1^**	**FeD^2^**	**Control^2^**	**FeH^2^**	***p*-value**
BW at 5-week-old (g)	18.43 ± 0.93	18.63 ± 1.86	17.91 ± 1.94	0.675
BW at 20-week-old (g)	43.60 ± 4.99^a^	34.51 ± 1.64^b^	36.98 ± 4.47^b^	< 0.001
BWG (g)	25.18 ± 5.01^a^	15.56 ± 2.78^b^	19.00 ± 3.41^ab^	< 0.001
Feed intake (g)	380.3 ± 18.1^a^	351.6 ± 24.2^b^	349.4 ± 21.9^b^	0.016
FI:BWG (g:g)	15.69 ± 3.40^b^	23.40 ± 5.51^a^	18.98 ± 4.06^ab^	0.010
Liver (g)	1.52 ± 0.21	1.37 ± 0.15	1.55 ± 0.23	0.141
Liver relative to BW (%)	4.58 ± 0.25	4.24 ± 0.28	4.80 ± 0.32	0.211
Epididymal fat (g)	2.04 ± 0.31^a^	1.24 ± 0.27^b^	2.03 ± 0.24^a^	0.003
Epididymal fat relative to BW (%)	6.30 ± 0.34^a^	5.07 ± 0.35^b^	6.25 ± 0.31^a^	0.011
Perirenal fat (g)	1.06 ± 0.14^a^	0.62 ± 0.15^b^	0.98 ± 0.136^a^	0.002
Perirenal fat relative to BW (%)	3.27 ± 0.23^a^	1.93 ± 0.25^b^	3.02 ± 0.21^a^	0.001
Retroepididymal fat (g)	1.03 ± 0.19	0.84 ± 0.14	0.89 ± 0.17	0.240
Retroepididymal fat relative to BW (%)	2.73 ± 0.29	2.59 ± 0.34	2.74 ± 0.52	0.431

^1^BW, body weight; BWG, body weight gain; FI: BWG, the ratio of feed intake and body weight gain.

^2^FeD—moderate iron deficient diet without Fe supplementation (containing 19.26 mg Fe/kg); Control—the FeD diet added with 200 mg Fe/kg, simulating the standard chow diet; FeH—the FeD diet added with 1,200 mg Fe/kg.

^a, b^Data are expressed as mean ± standard deviation (*n* = 10). Values within a column not sharing a common superscript letter differ significantly at *p* < 0.05.

### 3.2 Blood biochemical parameters

**Figure 1:**
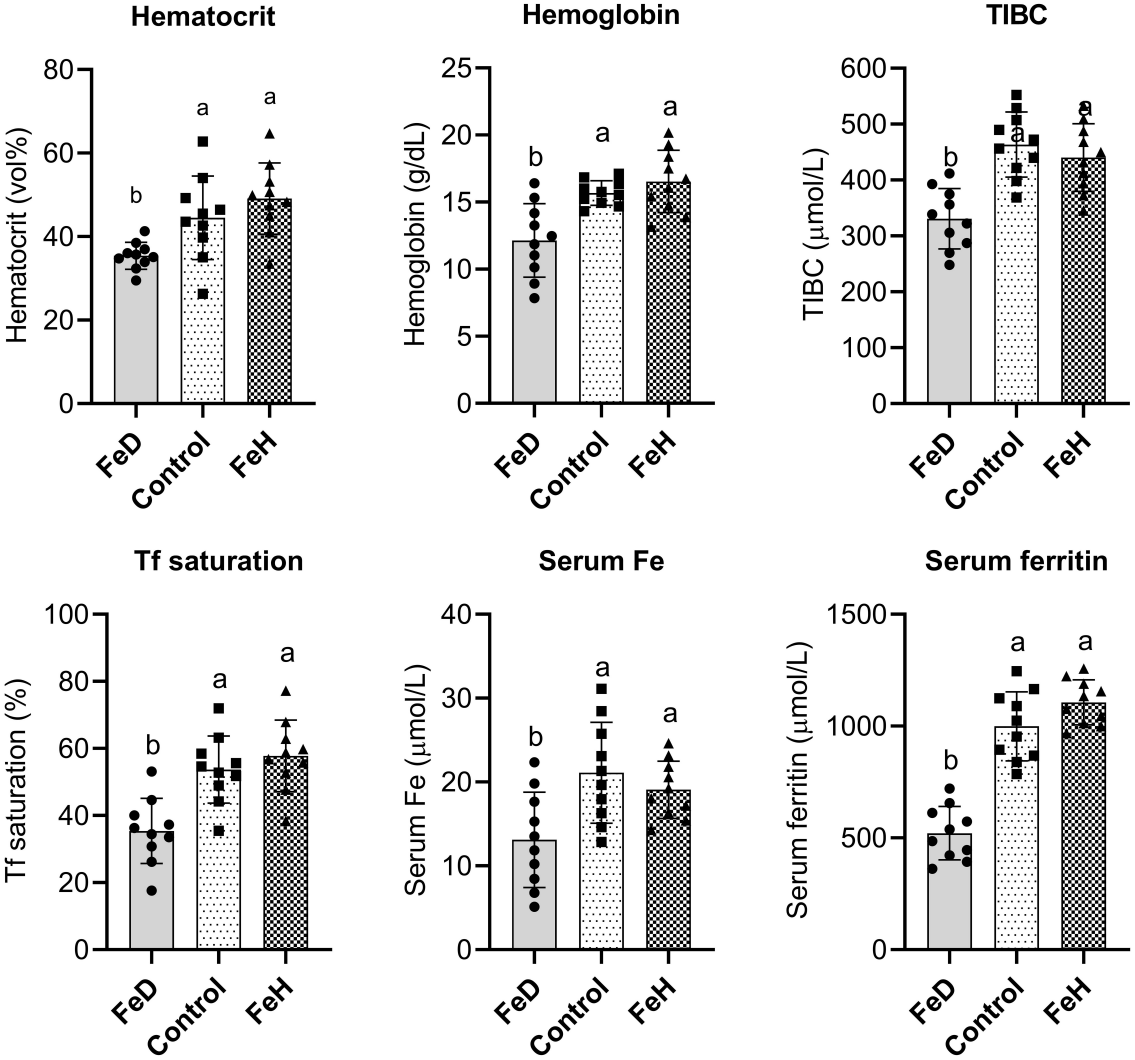
Dietary iron modulates hematological indicators of systemic iron status in male mice. TIBC, total iron-binding capacity; Tf, transferrin. FeD—moderate iron deficient diet without Fe supplementation (containing 19.26 mg Fe/kg); Control—the FeD diet added with 200 mg Fe/kg, simulating the standard chow diet; FeH—the FeD diet added with 1,200 mg Fe/kg. Data are expressed as mean ± standard deviation, with individual data points overlaid on the bars (*n* = 10). Bars not sharing a common lowercase letter are significantly different (*p* < 0.05) as determined by one-way ANOVA followed by Tukey's test.

FeD treatment also decreased blood Hb, serum TIBC, serum Fe, serum ferritin, and transferrin saturation compared with the control and FeH groups (*p* < 0.05; Figure 1), whereas hematocrit levels remained unaffected. The mean Hb level in the FeD group (12.8 ± 0.6 g/dL) remained above the established anemia threshold for C57BL/6J mice (< 12.0 g/dL) (28), indicating moderate iron deficiency without overt anemia. No significant differences in systemic Fe parameters were observed between the FeH and control groups.

**Figure 2:**
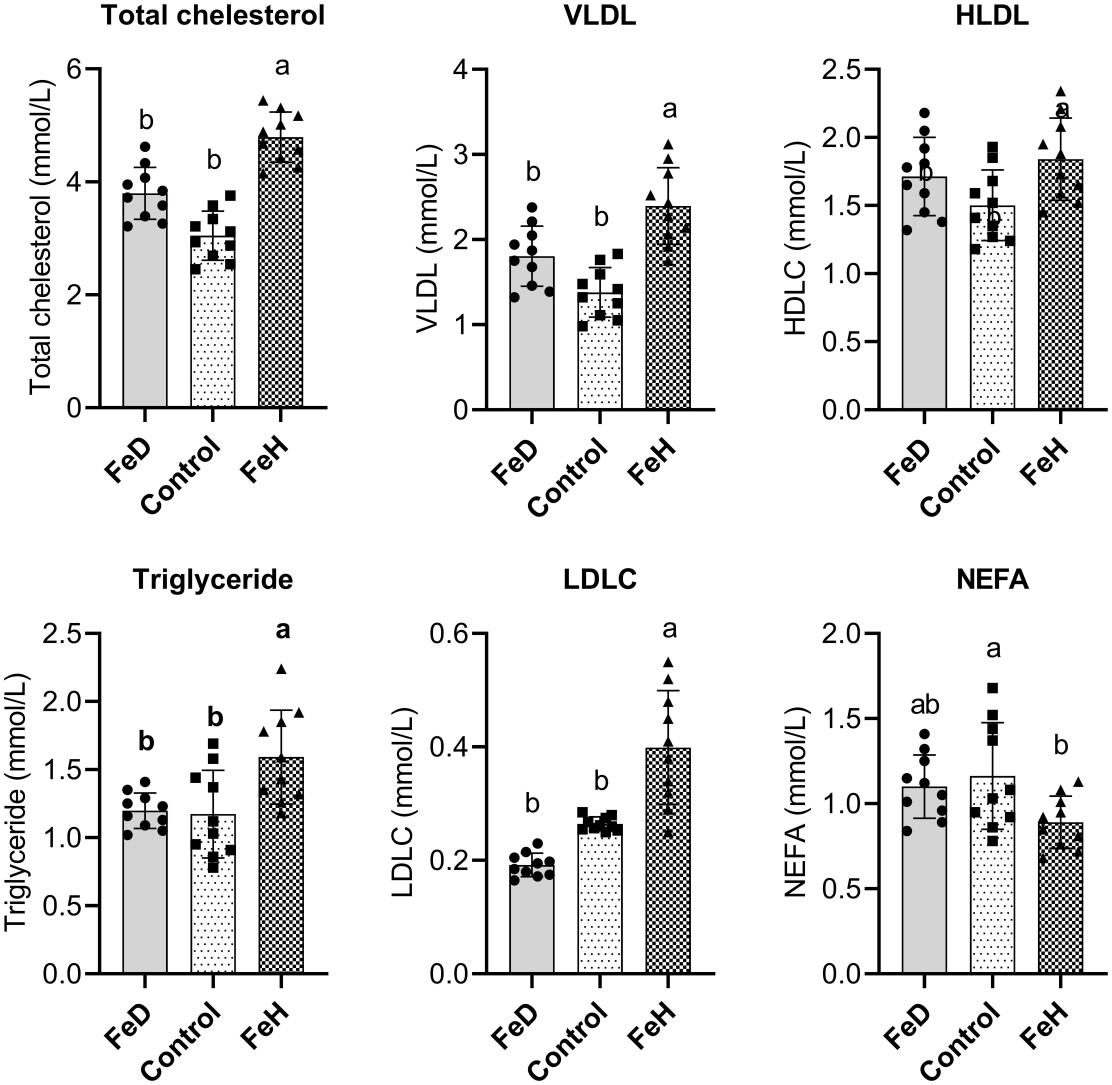
Effects of dietary iron level on lipid metabolic profiles in the plasma of male mice. VLDL, very low-density lipoprotein cholesterol; HDLC, high-density lipoprotein cholesterol; LDLC, low-density lipoprotein cholesterol; NEFA, non-esterified fatty acids. FeD—moderate iron deficient diet without Fe supplementation (containing 19.26 mg Fe/kg); Control—the FeD diet added with 200 mg Fe/kg, simulating the standard chow diet; FeH—the FeD diet added with 1,200 mg Fe/kg. Data are expressed as mean ± standard deviation, with individual data points overlaid on the bars (*n* = 10). Bars not sharing a common lowercase letter are significantly different (*p* < 0.05) as determined by one-way ANOVA followed by Tukey's test.

Regarding lipid metabolism, the FeH group showed significantly elevated serum concentrations of total cholesterol, triglycerides, VLDL, and LDLC relative to both the control and FeD groups (*p* < 0.05; Figure 2). In contrast, FeD did not significantly alter these lipid parameters compared with the control. Additionally, serum NEFA levels were significantly lower in the FeH group (*p* < 0.05), while FeD administration produced no significant change.

### 3.3 Fe deposition in tissues

**Figure 3:**
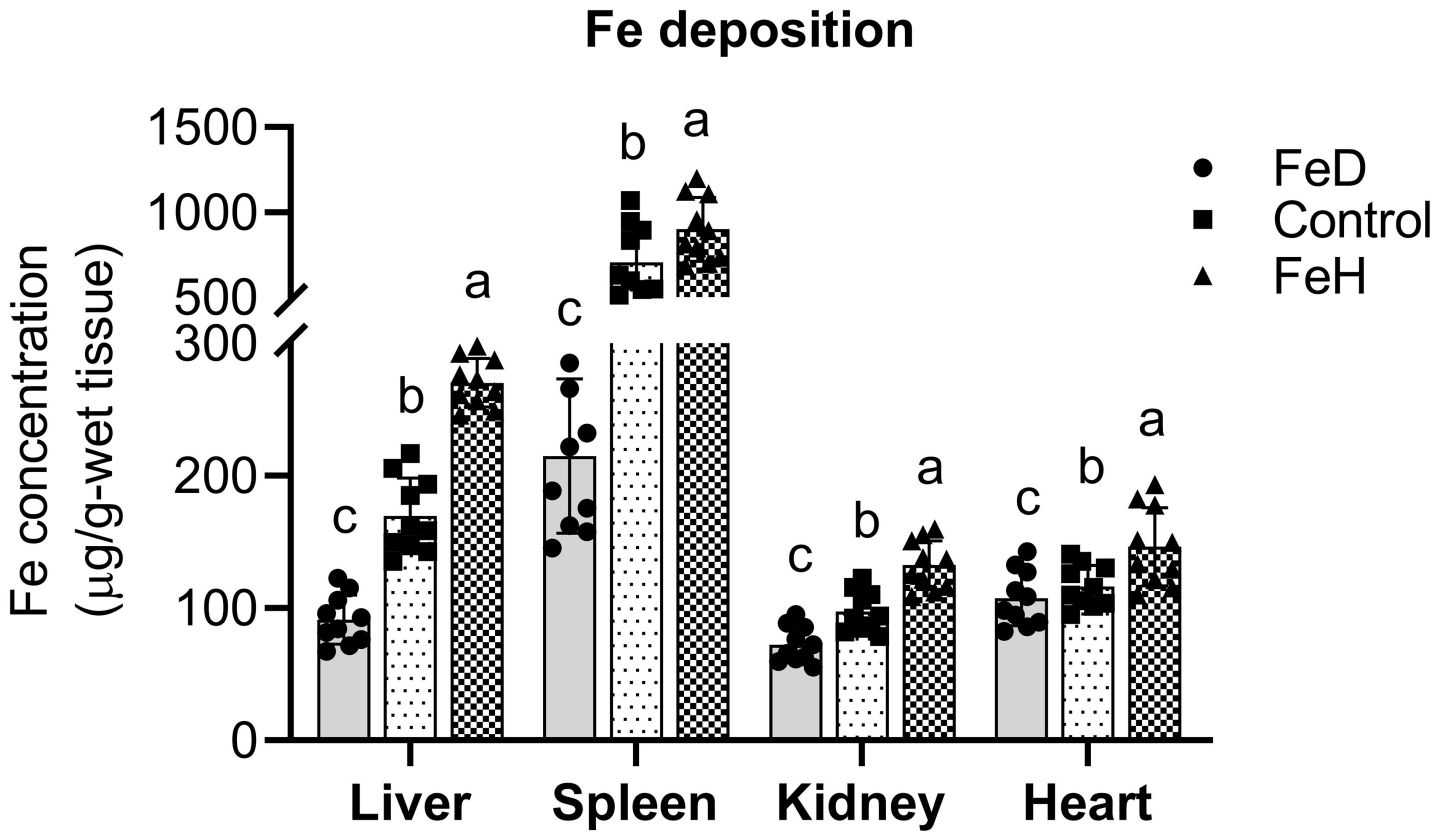
Dietary iron level influences the iron deposition in the different tissue of male mice. FeD—moderate iron deficient diet without Fe supplementation (containing 19.26 mg Fe/kg); Control—the FeD diet added with 200 mg Fe/kg, simulating the standard chow diet; FeH—the FeD diet added with 1,200 mg Fe/kg. Data are expressed as mean ± standard deviation, with individual data points overlaid on the bars (*n* = 10). Bars not sharing a common lowercase letter are significantly different (*p* < 0.05) as determined by one-way ANOVA followed by Tukey's test.

Compared with the control group, FeH treatment significantly increased Fe concentrations in liver, spleen, kidney, and heart (*p* < 0.05; Figure 3). In contrast, FeD administration markedly reduced Fe levels in the liver, spleen, and kidney, while no significant difference was observed in cardiac Fe content.

### 3.4 Hepatic triglyceride concentration and fatty acid profile

Hepatic triglyceride content was significantly lower in the FeH group compared with both the FeD and control groups (Table 2), a notable finding given the concurrent upregulation of lipogenic genes, whereas no significant difference was detected between the FeD and control groups. The biologically significant aspect of the hepatic TAG change in FeH is not its absolute magnitude but its directionality despite a pronounced lipogenic stimulus. Liver total cholesterol levels were not significantly affected by dietary Fe intervention.

**Table 2:** Dietary iron level modulates hepatic triglyceride accumulation and fatty acid profiles (g/100 fatty acids) in male mice.

**Items^1^**	**FeD^2^**	**Control^2^**	**FeH^2^**	***p*-value**
Triglyceride (mmol/g protein)^3^	30.10 ± 4.21^a^	29.76 ± 3.42^a^	21.61 ± 2.35^b^	0.040
Cholesterol (mmol/g protein)^3^	10.79 ± 1.30	9.70 ± 1.01	10.55 ± 1.23	0.702
Total fatty acids (mg/g wet-tissue)	306.6 ± 20.6^a^	214.5 ± 16.5^b^	353.0 ± 24.9^a^	< 0.001
C14:0	0.15 ± 0.07^ab^	0.24 ± 0.02^a^	0.13 ± 0.04^b^	0.023
C16:0	18.60 ± 2.0^b^	16.06 ± 2.1^b^	25.97 ± 1.7^a^	< 0.001
C18:0	12.91 ± 1.7^a^	5.51 ± 1.7^b^	4.89 ± 1.8^b^	< 0.001
C18:1 n-9	30.16 ± 3.5^b^	37.31 ± 2.9^a^	39.00 ± 2.9^a^	0.043
C18:2 n-6 (LA)	26.53 ± 1.9^a^	19.64 ± 2.3^b^	21.16 ± 1.9^b^	0.031
C18:3 n-3 (ALA)	2.70 ± 0.1^a^	1.29 ± 0.2^b^	0.88 ± 0.1^b^	< 0.001
C20:1 n-9	0.37 ± 0.05	0.66 ± 0.08	0.55 ± 0.12	0.094
C20:3 n-6	0.09 ± 0.03^b^	0.15 ± 0.06^a^	0.06 ± 0.03^b^	0.008
C20:4 n-6 (AA)	5.40 ± 0.6^b^	7.43 ± 0.8^a^	4.94 ± 0.7^b^	< 0.001
C20:5 n-3 (EPA)	0.13 ± 0.07	0.19 ± 0.09	0.13 ± 0.05	0.673
C22:6 n-3 (DHA)	2.98 ± 0.5^b^	5.74 ± 0.4^a^	2.54 ± 0.7^b^	0.001
SFAs	31.66 ± 5.2^a^	21.81 ± 4.1^b^	30.86 ± 4.2^a^	0.022
MUFAs	30.53 ± 3.6^b^	37.97 ± 3.3^ab^	39.55 ± 3.5^a^	0.025
PUFAs	37.81 ± 4.7^ab^	40.22 ± 4.1^a^	29.59 ± 3.8^b^	0.024
LCPUFA	8.60 ± 0.8^b^	13.51 ± 1.1^a^	7.67 ± 0.8^b^	< 0.001
C18:1 n-9/C18:0	2.35 ± 0.3^c^	6.77 ± 0.5^b^	7.98 ± 0.6^a^	< 0.001
C20:4 n-6/C18:2 n-6	0.20 ± 0.02^b^	0.38 ± 0.06^a^	0.23 ± 0.03^b^	< 0.001
(C20:5 n-3+C22:6 n-3)/C18:3 n-3	1.15 ± 0.2^c^	4.60 ± 0.6^a^	3.03 ± 0.4^b^	< 0.001
n-6/n-3 PUFA ratio	5.81 ± 0.4^b^	3.77 ± 0.5^c^	7.37 ± 0.5^a^	< 0.001
n-6/n-3 LCPUFA ratio	8.60 ± 0.5^b^	13.51 ± 1.0^a^	7.67 ± 0.6^b^	< 0.001

^1^SFAs correspond to C14:0, C16:0, and C18:0; MUFAs correspond to C18:1 n-9 and C20:1 n-9; PUFAs correspond to C18:2 n-6, C18:3 n-3, C20:3 n-6, C20:4 n-6, C20:4 n-6, C20:5 n-3, and C22:6 n-3; n-6 LCPUFAs are C20:3 n-6 and C20:4 n-6; n-3 LCPUFAs are C20:5 n-3 and C22:6 n-3; n-6/n-3 LCPUFA ratio represents (C20:3 n-6 and C20:4 n-6)/(C20:5 n-3+C22:6 n-3). LA, linoleic acid; ALA, a-linolenic acid; AA, arachidonic acid; EPA, eicosapentaenoic acid; DHA, docosahexaenoic acid; SFA, saturated fatty acid; MUFA, monounsaturated fatty acid; PUFA, polyunsaturated fatty acid; LCPUFA, long-chain polyunsaturated fatty acid.

^2^FeD—moderate iron deficient diet without Fe supplementation (containing 19.26 mg Fe/kg); Control—the FeD diet added with 200 mg Fe/kg, simulating the standard chow diet; FeH—the FeD diet added with 1,200 mg Fe/kg.

^3^Data expressed per mg protein to correct for variation in hepatic composition across dietary groups. Mean protein concentration was 175 mg/g wet tissue and did not differ significantly between groups (*p* > 0.05).

^a−c^Data are expressed as mean ± standard deviation (*n* = 10) and represent percentages of total fatty acids unless otherwise noted. Values within a column not sharing a common superscript letter differ significantly at *p* < 0.05.

Both FeD and FeH treatments led to a significant increase in total hepatic fatty acid (TFA) content, the relative proportion of saturated fatty acids (SFAs), and the n-6/n-3 PUFA ratio (*p* < 0.05). Conversely, both Fe-altered diets decreased the relative abundances of C20:3 n-6, C20:4 n-6, C22:6 n-3, and total long-chain PUFAs (LCPUFAs), along with reduced ratios of C20:4 n-6/C18:2 n-6, (C20:5 n-3 + C22:6 n-3)/C18:3 n-3, and n-6/n-3 LCPUFA compared to the control (*p* < 0.05).

Specifically, the FeD group showed elevated proportions of C18:0, C18:2 n-6, and C18:3 n-3, but a reduction in C18:1 n-9 and the C18:1/C18:0 ratio (*p* < 0.05). In comparison, the FeH group exhibited increased C16:0 and C18:1 n-9/C18:0 ratio, alongside decreased C14:0 and total PUFA levels (*p* < 0.05). Furthermore, the FeH group demonstrated significantly higher hepatic C16:0, total MUFAs, C18:1 n-9/C18:0 ratio, and n-6/n-3 PUFA ratio, but lower C18:0, C18:2 n-6, and C18:3 n-3 relative to the FeD group. No significant differences were detected between FeD and FeH groups in the relative proportions of C14:0, C20:4 n-6, C22:6 n-3, SFAs, PUFAs, or LCPUFAs.

### 3.5 Activity or protein expression of enzymes involved in lipid metabolism

**Figure 4:**
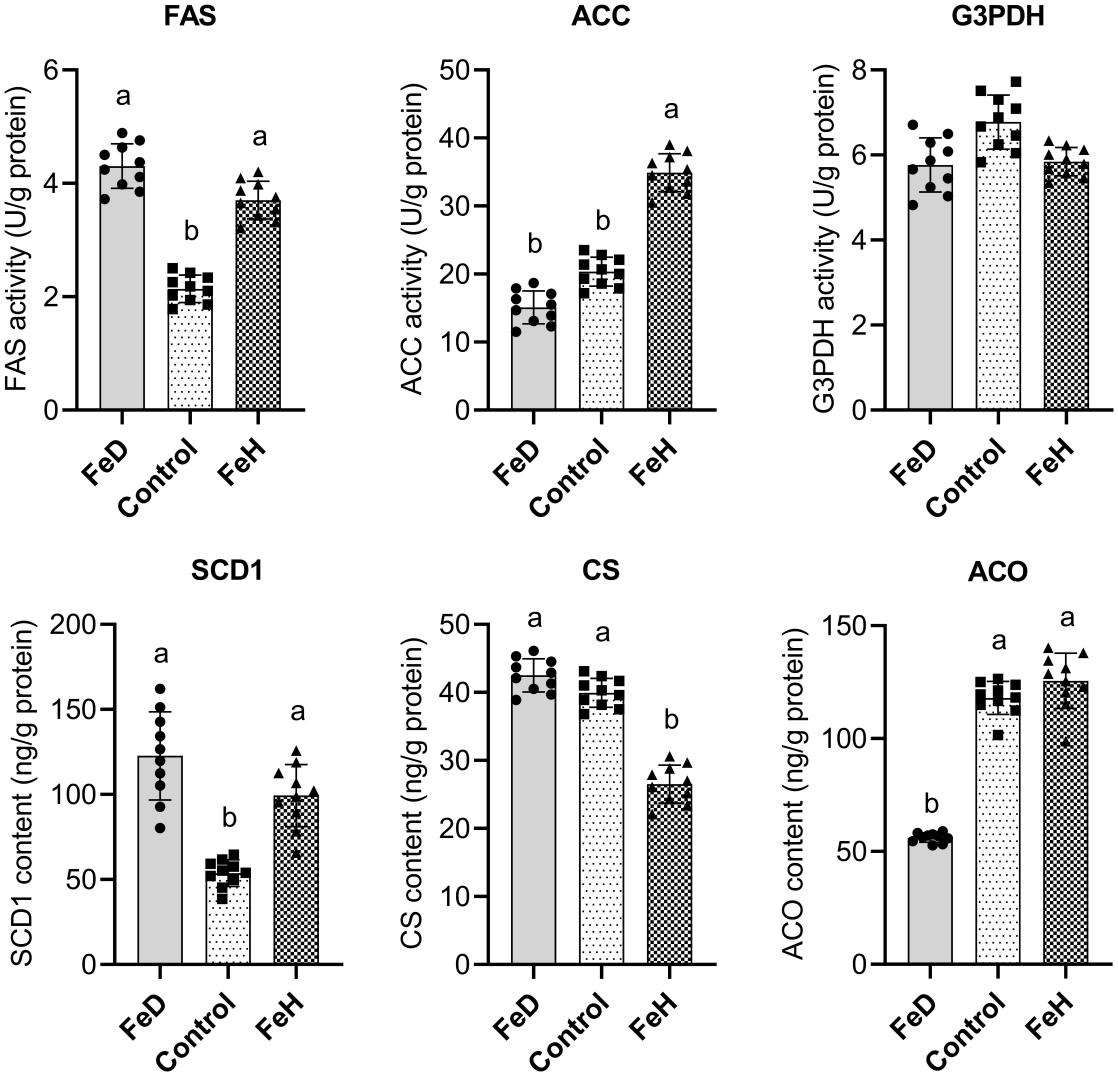
Effects of dietary iron level on hepatic enzymic activity or protein expression in male mice. FAS, fatty acid synthetase; ACC, acetyl-CoA carboxylase; G3PDH, glycerol-3-phosphate dehydrogenase; SCD1, stearoyl-CoA desaturase; CS, citrate synthetase; ACO, aconitase. FeD—moderate iron deficient diet without Fe supplementation (containing 19.26 mg Fe/kg); Control—the FeD diet added with 200 mg Fe/kg, simulating the standard chow diet; FeH—the FeD diet added with 1,200 mg Fe/kg. Data are expressed as mean ± standard deviation, with individual data points overlaid on the bars (*n* = 10). Bars not sharing a common lowercase letter are significantly different (*p* < 0.05) as determined by one-way ANOVA followed by Tukey's test with False Discovery Rate correction for multiple comparisons.

Both FeD and FeH treatments significantly increased hepatic FAS activity and SCD1 protein expression (*p* < 0.05; Figure 4). However, the functional *in vivo* Δ9-desaturase activity index (C18:1 n-9/C18:0 ratio) was significantly decreased in the FeD group (Table 2), indicating a post-translational functional impairment. In contrast, G3PDH activity remained unaffected by dietary Fe levels.

The FeH group exhibited significantly elevated ACC activity (*p* < 0.05) but reduced CS protein expression (*p* < 0.05; Figure 4). No significant changes in these parameters were observed in the FeD group. Additionally, hepatic ACO protein expression was significantly decreased in the FeD group (*p* < 0.05), whereas FeH treatment had no notable effect.

### 3.6 mRNA expression of enzymes or factors involved in lipid metabolism

**Figure 5:**
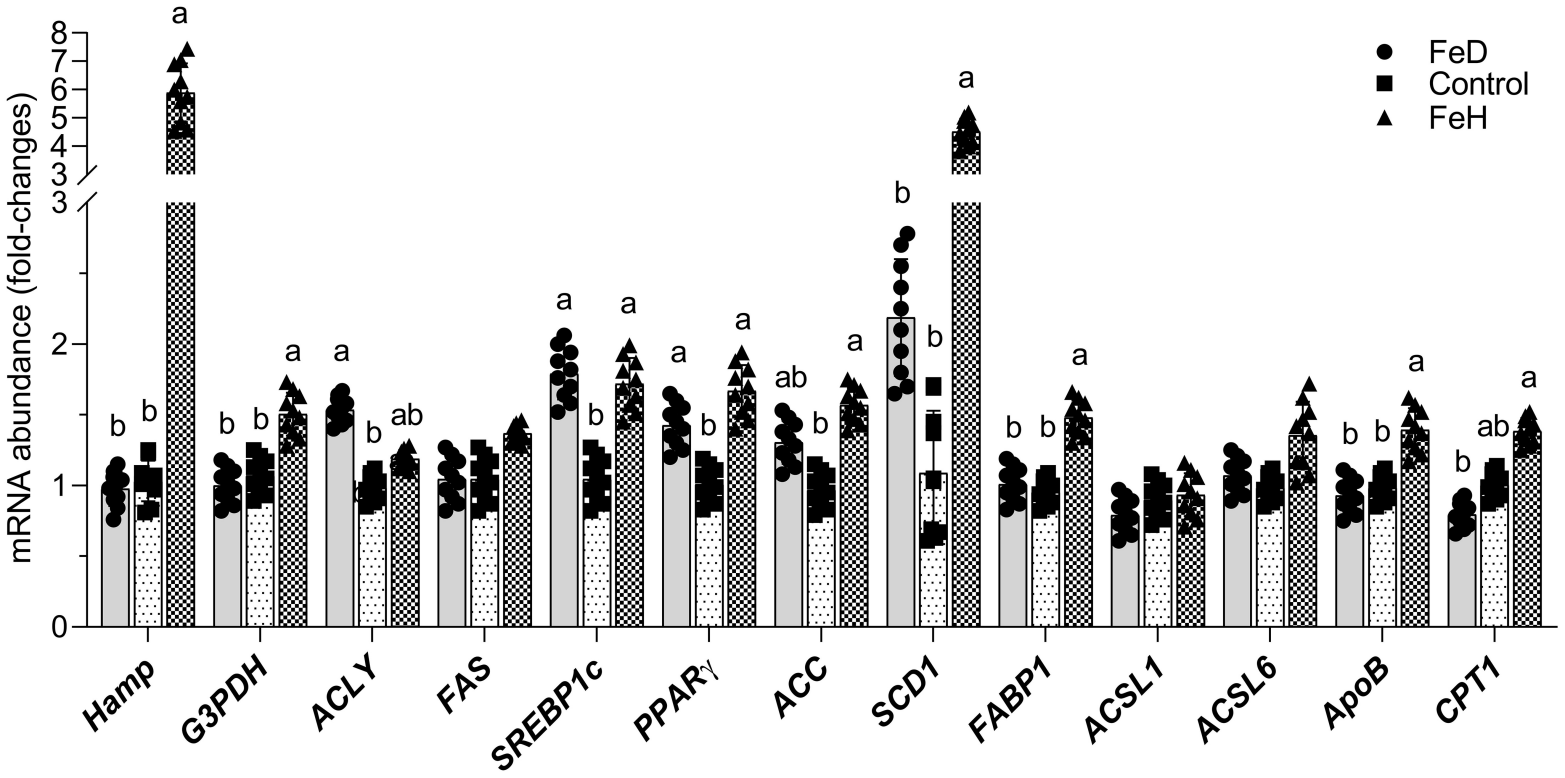
Effects of dietary iron on the mRNA abundance of key transcriptional regulators or enzymes involving lipid metabolism in the liver of male mice. G3PDH, glycerol-3-phosphate dehydrogenase; ACLY, ATP-citrate lyase; FAS, fatty acid synthetase; SREBP1c, sterol regulatory element-binding transcription factor 1c; PPARγ, peroxisome proliferator-activated receptor-γ; ACC, acetyl-COA carboxylase; SCD1, stearoyl-COA desaturase; FABP1, fatty acid-binding protein 1; ACSL, long-chain acyl-CoA synthetase; ApoB, apolipoprotein B; CPT, carnitine palmitoyl acyl-CoA transferase. FeD—moderate iron deficient diet without Fe supplementation (containing 19.26 mg Fe/kg); Control—the FeD diet added with 200 mg Fe/kg, simulating the standard chow diet; FeH—the FeD diet added with 1,200 mg Fe/kg. Data are expressed as mean ± standard deviation, with individual data points overlaid on the bars (*n* = 10). Bars not sharing a common lowercase letter are significantly different (*p* < 0.05) as determined by one-way ANOVA followed by Tukey's test with False Discovery Rate correction for multiple comparisons.

Hepatic hepcidin (*Hamp*) mRNA expression was significantly upregulated in the FeH group compared to control and FeD groups (*p* < 0.05), while FeD treatment did not alter *Hamp* expression (Figure 5). Dietary Fe levels significantly modulated the transcriptional expression of several genes related to lipid metabolism, with the exception of *FAS*, long-chain acyl-CoA synthetase (*ACSL)1*, and *ACSL6* in the liver of mice (Figure 5). The FeH group showed significantly upregulated mRNA abundance of *G3PDH, SREBP1c, PPAR*γ, *ACC, SCD1, FABP1, ApoB*, and *CPT1* (*p* < 0.05) in the liver, compared with the control group (Figure 5). No significant change was observed in hepatic *ACLY* mRNA expression between the FeH and control groups. In contrast, the FeD treatment significantly increased the mRNA abundance of *ACLY, SREBP1c*, and *PPAR*γ (*p* < 0.05), but did not significantly affect the mRNA expression of *G3PDH, ACC, SCD1, FABP1, ApoB*, or *CPT1* in the liver.

**Figure 6:**
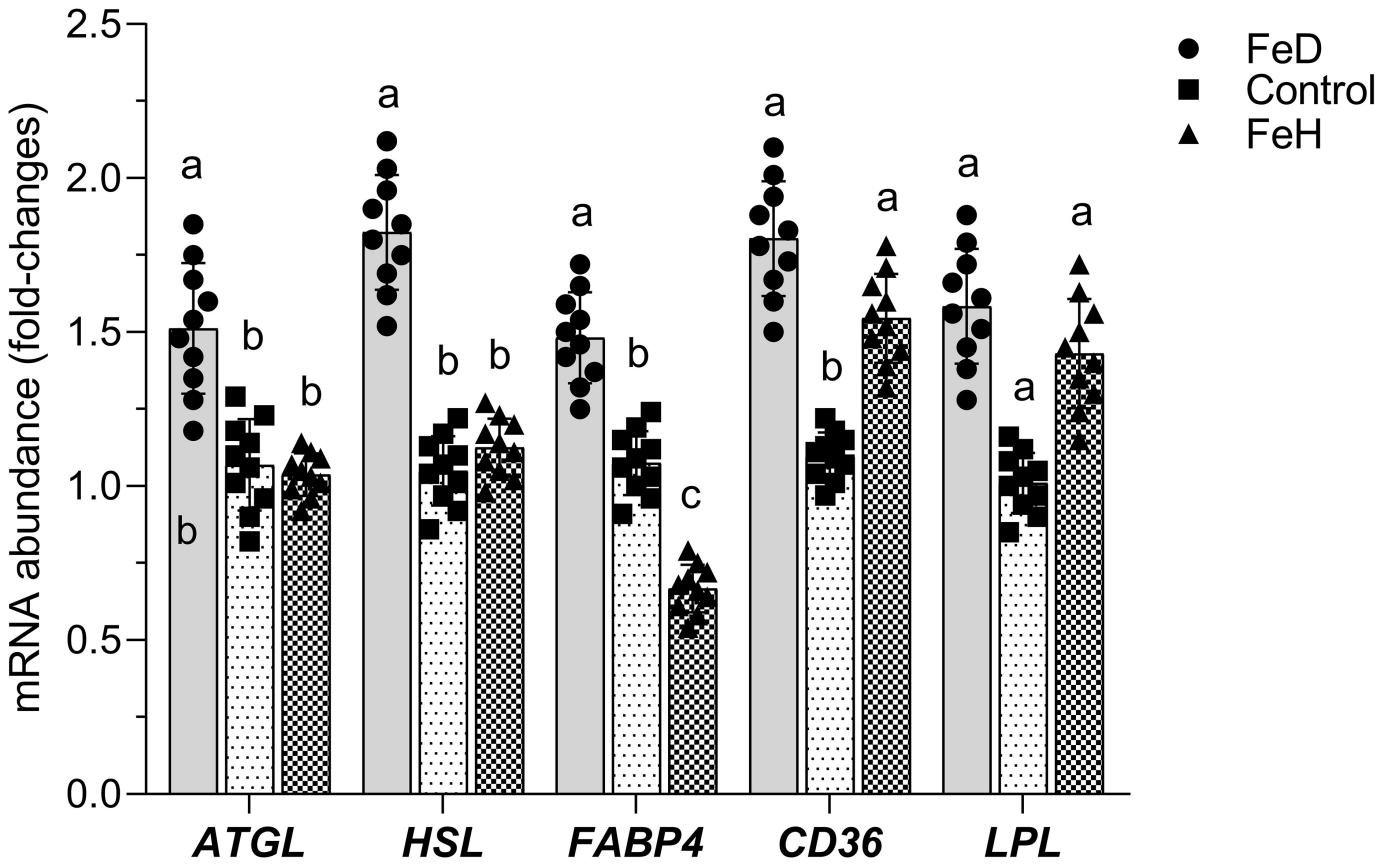
Effects of dietary iron on the mRNA abundance of key regulators or enzymes involving lipid metabolism in the epididymal adipose tissue of male mice. ATGL, adipose triglyceride lipase; HSL, hormone-sensitive lipase; FABP1, fatty acid-binding protein 1; CD36, the cluster of differentiation 36; LPL, lipoprotein lipase. FeD—moderate iron deficient diet without Fe supplementation (containing 19.26 mg Fe/kg); Control—the FeD diet added with 200 mg Fe/kg, simulating the standard chow diet; FeH—the FeD diet added with 1,200 mg Fe/kg. Data are expressed as mean ± standard deviation, with individual data points overlaid on the bars (*n* = 10). Bars not sharing a common lowercase letter are significantly different (*p* < 0.05) as determined by one-way ANOVA followed by Tukey's test with False Discovery Rate correction for multiple comparisons.

In epididymal adipose tissue, both FeH and FeD treatments significantly elevated the mRNA levels of *CD36* and *LPL* compared with the control group (*p* < 0.05; Figure 6). The FeD group also showed increased transcriptional expression of *HSL, ATGL*, and *FABP4* (*p* < 0.05). In contrast, FeH administration decreased *FABP4* mRNA levels (*p* < 0.05) and had no significant effect on *ATGL* or *HSL* expression.

## 4 Discussion

This study demonstrates that both moderate Fe deficiency (FeD) and high dietary Fe (FeH) disrupt hepatic lipid metabolism through divergent pathways: FeD primarily induces systemic adaptations and alters fatty acid handling, whereas FeH elicits direct hepatic lipogenic activation but ultimately reduces triglyceride accumulation. While both dietary iron manipulations elevated total hepatic fatty acids, their functional outcomes were fundamentally different.

Our finding that high dietary iron (FeH) significantly increased hepatic hepcidin (*Hamp*) expression provides a potential link between iron overload and the observed lipogenic reprogramming. Hepcidin is not only central to iron homeostasis but is also regulated by and may influence metabolic processes (29). Recent studies have shown that lipogenic transcription factors such as *SREBP*-1 can activate hepcidin expression, revealing a feed-forward loop between lipid synthesis and iron regulation (30). Furthermore, aberrant hepatocyte iron distribution mediated by the hepcidin-ferroportin axis can drive hepatic lipogenesis (31). The concurrent upregulation of *Hamp* and key lipogenic genes (*SREBP1c, ACC, FAS*) in our FeH model supports the existence of such cross-talk, suggesting that elevated hepcidin may be both a consequence and a contributor to the iron-overload-induced hepatic metabolic shift relevant to a subset of NAFLD.

In the present study, high Fe feeding (FeH) did not significantly affect BW of mice, consistent with previous reports using moderate Fe-overloaded diets (15, 27). In contrast, moderate Fe deficiency (FeD) increased both BW and feed intake. This increase in BW is likely a direct consequence of the observed hyperphagia. This contrasts with studies using more severe Fe-deficient diets (32), suggesting that the metabolic consequences of Fe deficiency are highly dependent on its severity. The observed hyperphagia in FeD mice indicates a compensatory behavioral adaptation to meet Fe demands through increased consumption. These divergent outcomes across studies likely arise from differences in Fe concentration, treatment duration, animal model, and basal diet composition.

We utilized a moderate Fe-deficient diet to better mimic common human clinical presentations. After 16 weeks, FeD mice exhibited characteristic declines in systemic Fe parameters (Hb, serum Fe, ferritin, TIBC, and transferrin saturation). The high-Fe diet (FeH) did not further elevate these circulatory markers compared to the adequate-Fe control, supporting the concept of tightly regulated systemic Fe homeostasis. However, FeH significantly increased Fe deposition in the liver and spleen. This aligns with the established model of the liver as a primary storage organ for excess Fe (33). The splenic Fe accumulation is likely attributable to enhanced erythrophagocytosis and Fe recycling by splenic macrophages (34, 35), highlighting the role of the spleen in Fe recycling under overload conditions. It is noteworthy that the FeD mice in this study exhibited moderate iron deficiency without crossing the threshold into anemia (Hb ≥ 12.0 g/dL) (28). This model therefore reflects the metabolic alterations specific to iron restriction, distinct from those driven by anemic hypoxia or severe erythropoietic stress. Future studies comparing anemic and non-anemic iron deficiency models could further dissect the contributions of iron lack vs. oxygen delivery to lipid metabolic dysregulation.

Moderate Fe deficiency did not alter serum cholesterol or triglyceride levels, a finding consistent with observations in both moderately Fe-deficient rats and humans (36, 37). In contrast, FeH feeding may contribute to induced marked hyperlipidemia, characterized by elevated plasma triglycerides and cholesterol—a result corroborated by other studies using Fe-enriched diets (27, 38). The observed hyperlipidemia in FeH mice coincided with transcriptional upregulation of hepatic *de novo* lipogenesis genes (*SREBP1c, ACC, FAS, SCD1*) and reduced serum NEFA levels. The reduction in plasma NEFA observed in FeH mice suggests suppressed peripheral lipolysis. The observed hyperlipidemia in FeH mice therefore likely stem primarily from enhanced hepatic *de novo* lipogenesis rather than increased lipid mobilization from adipose tissue, though direct flux measurements are needed for confirmation.

The decrease in hepatic triglyceride (TAG) content in the FeH group, despite clear activation of lipogenic pathways, presents a biologically significant paradox. The biologically significant aspect is not the absolute magnitude of TAG change but its directionality despite a pronounced lipogenic stimulus. This indicates a potent compensatory shift in hepatic lipid fate toward oxidation or secretion, as suggested by elevated *CPT1* and *ApoB* expression. Similar dissociations between induced lipogenesis and TAG accumulation have been reported in iron-overloaded rodents, where compensatory increases in β-oxidation and VLDL secretion appear to mitigate hepatic steatosis (18, 39). Additionally, iron overload is known to promote lipid peroxidation and shift lipid metabolism toward pathways such as ferroptosis, which may alter lipid composition without necessarily increasing TAG storage (40, 41). In contrast, the FeD group displayed altered hepatic fatty acid composition—characterized by increased saturation and modified PUFA ratios—without a concomitant rise in TAG content. This pattern suggests a remodeling of hepatic lipid species rather than the classical lipid accumulation seen in steatosis, a phenomenon also reported in moderately iron-deficient rodent models (36). While our transcriptional and lipidomic data support these interpretations, future studies directly measuring fatty acid oxidation rates and VLDL secretion would be valuable to confirm these proposed mechanisms and further elucidate the distinct hepatic lipid handling strategies under conditions of iron excess and deficiency.

The most compelling insight from this study is that at the examined points on the iron status spectrum—moderate deficiency and high intake—hepatic lipid metabolism is disrupted via clearly divergent pathways. The FeH group exhibited a coherent transcriptional activation program, driven by *SREBP1c*, leading to the coordinated upregulation of its lipogenic targets (*ACC, FAS*, and *SCD1*). In stark contrast, the FeD group showcased a major post-translational constraint. The most revealing finding was the disconnect between elevated SCD1 protein and decreased functional output (C18:1/C18:0 ratio). SCD1 is an iron-dependent enzyme whose catalytic activity requires the incorporation of iron cofactors (42, 43). The U-shaped pattern of SCD1 protein (high in both FeD and FeH) likely reflects distinct regulatory mechanisms: transcriptional upregulation via *SREBP1c* in FeH, and a possible post-transcriptional response in FeD that may represent a compensatory increase in protein synthesis despite functional iron lack. Importantly, the upregulation of lipogenic genes in FeD mice may be confounded by compensatory hyperphagia and the resultant caloric surplus. In the absence of a pair-fed control, we cannot definitively dissociate the direct molecular effects of iron deficiency from the secondary effects of increased energy intake. Therefore, the FeD lipogenic phenotype is best interpreted as a holistic physiological adaptation to moderate iron deficiency, and future studies employing pair-feeding are essential.

A key finding was that both Fe dysregulation states were associated with altered mitochondrial enzyme protein levels. FeD decreased hepatic aconitase (ACO) protein, indicating potential impairment of Fe–S cluster integrity (28). FeH reduced CS protein, commonly interpreted as a reduction in mitochondrial content (44, 45). While this suggests a diminished mitochondrial metabolic capacity under Fe overload, we acknowledge that without direct measurements of oxidative phosphorylation or respiratory chain function, the precise nature of the functional impairment remains to be fully elucidated.

In this study, both FeD and FeH upregulated mRNA expression of *CD36* and *LPL* in epididymal adipose tissue, suggesting increased capacity for fatty acid uptake from circulation. However, a stark divergence was observed in lipolytic regulation. Only FeD significantly increased the expression of key lipolytic enzymes (*ATGL* and *HSL*) and the lipid shuttle *FABP4*. This indicates that moderate Fe deficiency specifically enhances adipose tissue lipolysis, releasing fatty acids that may contribute to hepatic lipid accumulation. The lack of such an effect in FeH mice further emphasizes the model-specific pathways leading to steatosis.

We note that the FeD mice in this study exhibited moderate iron deficiency without crossing the threshold into anemia. This model therefore reflects the metabolic alterations specific to iron restriction, distinct from those driven by anemic hypoxia. A major limitation in interpreting our iron-deficient (FeD) model is the compensatory hyperphagia, which confounds the specific effects of iron deficiency. Although we observed upregulation of hepatic lipogenic genes and enzymes, this phenotype may be secondary to increased energy intake rather than a direct consequence of low iron. The absence of a pair-fed control prevents definitive dissociation of iron-specific molecular effects from caloric surplus. Future studies must include pair-fed groups to isolate the primary targets of iron deficiency from its metabolic consequences. Furthermore, we assessed the protein levels of SCD1, CS, and ACO rather than their enzymatic activities. While protein abundance provides valuable information, direct activity measurements in future studies could offer more precise insights into functional insights. While our sample size provided sufficient power for the primary outcomes, future investigations exploring more subtle or interactive effects may benefit from larger cohorts. The use of targeted fatty acid profiling, while informative, captures only one dimension of the lipidome. Future studies employing untargeted lipidomics could reveal critical changes in specific lipid classes (e.g., diacylglycerols, ceramides, phospholipid species) that may mediate the metabolic effects of iron dysregulation. Future studies employing untargeted transcriptomic approaches could also uncover additional iron-sensitive pathways in lipid metabolism.

## 5 Conclusions

The most compelling insight from this study is that both dietary Fe excess and moderate iron deficiency disrupt hepatic lipid metabolism via clearly divergent pathways: FeH through direct transcriptional activation of hepatic lipogenesis, potentially involving hepcidin-mediated cross-talk, and FeD through systemic adaptations leading to post-translational constraints on iron-dependent enzymes. These findings underscore the critical importance of maintaining iron homeostasis for metabolic health and provide a mechanistic framework for understanding the heterogeneous role of iron in conditions like NAFLD. Potential biomarker such as SCID activity and adipose lipolysis markers may help identify individuals with iron-related metabolic dysfunction, guiding personalized nutritional strategies for at-risk populations.

## Data Availability

The original contributions presented in the study are included in the article/Supplementary material, further inquiries can be directed to the corresponding author.
