# Supplementary material for: Moderate iron deficiency and high dietary iron intake differentially alter hepatic lipid metabolism and adipose tissue lipid handling in mice
Source: Front Nutr. 2026 Jan 27;12:1725052. doi: 10.3389/fnut.2025.1725052 (PMC12888867; doi:10.3389/fnut.2025.1725052)
Supplement: Supplementary file 1 [file Table_1.docx]

**Supplementary Table 1.** Composition and nutrient level of the basal diet (as-fed basis)

| Items | Percentage (%) |
| --- | --- |
| Ingredients |  |
| Corn starch | 39.05 |
| Casein | 21.75 |
| Maltodextrin | 13.30 |
| Sucrose | 10.00 |
| Soybean oil | 7.10 |
| *L*-cystine | 0.30 |
| Cellulose | 5.00 |
| Choline chlorides | 0.20 |
| Mineral mix^1^ | 3.20 |
| Vitamin mix^2^ | 0.10 |
| Calculated Nutrient levels |  |
| Metabolizable energy (kcal/kg) | 3900 |
| Crude protein | 18.30 |
| Crude fat | 7.10 |
| Fiber | 5.00 |
| Carbohydrate | 63.20 |
| Iron^3^ (mg/kg) | 19.26 |

^1^Provided per kg of complete diet: 5000 mg Ca (CaCO_3_, 40.04% Ca), 2000 mg P (potassium phosphate, containing 22.76% P and 28.73% K), 1000 Na (NaCl, containing 39.34% Na and 60.66% Cl), 1600 mg K (potassium citrate), 700 Mg (MgO), 6 mg Cu (CuSO_4_·5H_2_O), 10 mg Mn (MnSO_4_•H_2_O), 35 mg Zn (ZnSO_4_•H_2_O), 0.21 I (KI), 0.15 Se (Na_2_SeO_3_).

^2^Provided per kg of complete diet: 9000 IU vitamin A, 1500 IU vitamin D_3_, 75 IU vitamin E, 6 mg vitamin B_1_, 6 mg vitamin B_2_, 1.5 mg vitamin B_6_, 7.5 mg vitamin B_12_, 0.15 mg folic acid 0.15mg, 35 mg nicotinic acid.

^3^Analyzed value.

**Supplementary Table 2.** Key specifications and validation data of the commercial kits used for the analysis of systemic iron and lipid profiles.

| Analyte^1^ | Kit Name (Manufacturer) | Catalog No. | Assay type | Key reagents | Assay range and unit | LOD^2^ | LOQ^2^ | Specificity | Coefficient of variation (%) | Spike recovery (%) |
| --- | --- | --- | --- | --- | --- | --- | --- | --- | --- | --- |
| Serum iron | Serum iron content assay kit (Nanjing Jiancheng Bioengineering Institute, Nanjing, China) | A039-1-1 | Colorimetric | 2,2'-bipyridine, sodium sulfite, Detergent | 0.6-25 μmol/L | 0.2 μmol/L | 0.6 μmol/L | Validated for serum and plasma (human, mouse, and rat) | <3.0 | 95 |
| UIBC | Serum UIBC assay kit (Nanjing Jiancheng Bioengineering Institute, Nanjing, China) | A040-1-1 | Colorimetric | N-nitro-5-(N-propyl-N-sulfonylpropylamine)-phenol, ammonium ferric sulfate | 3-200 μmol/L | 1 μmol/L | 3 μmol/L | Validated for serum and plasma (human, mouse, and rat) | <3.0 | 95 |
| Serum ferritin | Mouse ferritin ELISA kit (Bioleaf Biotech, Shanghai, China) | 80636 | ELISA | Coated Antibody, HRP conjugate | 12.5-400 ng/mL | 0.5 ng/mL | 2.6 ng/mL | Specific for mouse ferritin | <8.0 | 95 |
| Total cholesterol | Total cholesterol assay kit (Nanjing Jiancheng Bioengineering Institute, Nanjing, China) | A111-1-1 | Enzymatic colorimetric | Cholesterol esterase, cholesterol oxidase, catalase, 4-aminoaminopyridine, sodium azide, N-(2-Hydroxy-3-sulfopropyl)-3,5-dimethoxyaniline | 0.6-19.39 mmol/L | 0.2 mmol/L | 0.6 mmol/L | Validated for serum and tissue (human, mouse, and rat) | <3.0 | 95 |
| HDLC | HDL cholesterol assay kit (Nanjing Jiancheng Bioengineering Institute, Nanjing, China) | A112-1-1 | Enzymatic colorimetric | Cholesterol esterase, cholesterol oxidase, sodium azide | 0.50-6.68 mmol/L | 0.13 mmol/L | 0.50 mmol/L | Validated for serum and plasma (human, mouse, and rat) | <2.0 | 95 |
| LDLC | LDL cholesterol assay kit (Nanjing Jiancheng Bioengineering Institute, Nanjing, China) | A113-1-1 | Enzymatic colorimetric | 4-aminoaminopyridine, N-Ethyl-N-(2-hydroxy-3-thiopropyl)-3-methylaniline, 1,4-piperazine diethylsulfonate, sodium azide | 0.3-25.9 mmol/L | 0.1 mmol/L | 0.3 mmol/L | Validated for serum and plasma (human, mouse, and rat) | <2.0 | 90 |
| NEFA | NEFA ELISA kit (Cell Biolabs, Inc., Beijing, China) | STA-619 | ELISA | Detergent, acyl CoA synthetase, acyl CoA oxidase | 7.81-500 μmol/L | 2 μmol/L | 6 μmol/L | Validated for serum and tissue (mouse, and rat) | <5.0 | 95 |
| TAG | Triglyceride assay kit (Nanjing Jiancheng Bioengineering Institute, Nanjing, China) | A110-1-1 | Colorimetric | 4-aminopyridine, parachlorphenol | 0.3-11.4 mmol/L | 0.1 μmol/L | 0.3 μmol/L | Validated for serum and tissue (mouse, and rat) | <10 | 96 |

^1^UIBC = Unsaturated iron-binding capacity; HDLC = High-density lipoprotein cholesterol; LDLC = Low-density lipoprotein cholesterol; NEFA = Nonesterified fatty acid; TAG = Triglyceride.

^2^LOD = Limit of detection; LOQ = limit of quantitation.

**Supplementary Table 3.** Key specifications and validation data of the commercial kits used for the analysis of enzyme activity or protein.

| Analyte^1^ | Kit Name (Manufacturer) | Catalog No. | Assay type | Key reagents | Assay range and unit | LOD^2^ | LOQ^2^ | Specificity and validation data | Coefficient of variation (%) | Assay principle |
| --- | --- | --- | --- | --- | --- | --- | --- | --- | --- | --- |
| FAS activity | FAS activity assay kit (Nanjing Jiancheng Bioengineering Institute, China) | H231-1-1 | Enzymatic | Acetyl-CoA, malonyl-CoA, NADPH | 10-1500 U/L | 3 U/L | 10 U/L | Validated for tissue homogenates (mammalian) | <10.0 | Spectrophotometric, monitoring NADPH consumption. |
| ACC activity | ACC activity assay kit (Nanjing Jiancheng Bioengineering Institute, China) | H232-1-1 | Enzymatic | Acetyl-CoA, NaHCO_3_, phosphoenolpyruvic acid, pyruvate kinase, lactate dehydrogenase, NADH | 6-150 U/L | 2 U/L | 6 U/L | Validated for tissue homogenates (mammalian) | <8.0 | Spectrophotometric, coupled reaction monitoring NADH consumption. |
| G3PDH activity | Glycerol-3-phosphate dehydrogenase (G3PDH) assay kit (Abcam plc, Cambridge, UK) | Ab174095 | Enzymatic | Tetrazolium salt, NADH | 3-80 U/L | 1 U/L | 3 U/L | Validated for tissue homogenates (mammalian) | <10.0 | Spectrophotometric, monitoring NAD⁺ reduction. |
| SCD1 protein concentration | Mouse SCD-1 ELISA kit (Shanghai Coibo Biological Company, Shanghai, China) | CB11924-Mu | ELISA | Detergent, 3,3',5,5'-tetramethylbenzidine, HRP-conjugated secondary antibody | 31.25-1000 pg/mL | 1 pg/mL | 5 pg/mL | Validated for mouse tissue lysates | <10.0 | Coated anti-SCD1 antibody → HRP-detection antibody → TMB substrate |
| CS protein concentration | Mouse CS ELISA kit (Shanghai Coibo Biological Company, Shanghai, China) | CB12662-Mu | ELISA | Detergent, 3,3',5,5'-tetramethylbenzidine, HRP-conjugated secondary antibody | 6.25-200 mg/L | 1 mg/L | 3 mg/L | Validated for mouse tissue lysates | <10.0 | Coated anti-CS antibody → HRP-detection antibody → TMB substrate |
| ACO protein concentration | Mouse ACO ELISA kit (Shanghai Coibo Biological Company, Shanghai, China) | CB12626-Mu | ELISA | Detergent, 3,3',5,5'-tetramethylbenzidine, HRP-conjugated secondary antibody | 12.5-400 mg/L | 1 mg/L | 3 mg/L | Validated for mouse tissue lysates | <10.0 | Coated anti-ACO antibody → HRP-detection antibody → TMB substrate |

^1^FAS = fatty acid synthase; ACC = Acetyl-CoA carboxylase; glycerol-3-phosphate dehydrogenase; SCD1 = stearoyl-CoA desaturase; ACO = aconitase; CS = citrate synthase;

^2^LOD = Limit of detection; LOQ = limit of quantitation.

**Supplementary Table 4.** The oligonucleotide sequences of the sense and antisense primers for real-time PCR products.

| Gene^1^ | Primer sequence (5’-3’) | GeneBank No. |
| --- | --- | --- |
| *Hamp* | F: GCACCACCTATCTCCATCAACA | NM_032541.2 |
|  | R: TTCTTCCCCGTGCAAAGG |  |
| *FABP1* | F: GCTGCGGCTGCTGTATGA | NM_017399.5 |
|  | R: CACCGGCCTTCTCCATGA |  |
| *G3PDH* | F: TACCTGCCAGGGCACAAG | NC_000081.7 |
|  | R: GGGTACCACAAAAACCAGGA |  |
| *SREBP1c* | F: GGAGCCATGGATTGCACATT | AF374266.1 |
|  | R: GGCCCGGG AAGTCACTGT |  |
| *PPARγ* | F: CACCAGTGTGAATTACAGCAAATC | NR_176971.1 |
|  | R: ACAGGAGAATCTCCCAGAGTTTC |  |
| *ACC* | F: TGACAGACTGATCGCAGAGAAAG | AF289714.1 |
|  | R: TGGAGAGCCCCACACACA |  |
| *FAS* | F: GGAGGTGGTGATAGCCGGTAT | NM_007988.3 |
|  | R: TGGGTAATCCATAGAGCCCAG |  |
| *ACLY* | F: ACCCTTTCACTGGGGATCACA | NM_001199296.1 |
|  | R: GACAGGGATCAGGATTTCCTTG |  |
| *SCD1* | F: CCGGAGACCCCTTAGATCGA | AH002082.2 |
|  | R: TAGCCTGTAAAAGATTTCTGCAAACC |  |
| *CPT1* | F: TGGCATCATCACTGGTGTGTT | NM_009948 |
|  | R: GTCTAGGGTCCGATTGATCTTTG |  |
| *ApoB* | F: CGTGGGCTCCAGCATTCTA | NM_009693.2 |
|  | R: TCACCAGTCATTTCTGCCTTTG |  |
| *ACSL1* | F: CGTGCGTGACATCAAAGAGAA | BC056644.1 |
|  | R: CCAAGAAGGAAGGCTGGAAAA |  |
| *ACSL6* | F: ACGAGGACAGGACAAAGGAG | NM_144823 |
|  | R: CTCTGG CGCAACATATTCCC |  |
| *ATGL* | F: TGGAACTGTCTCGTGGG | AY894805.1 |
|  | R: CTACTGTGGGCTGATACCT |  |
| *HSL* | F: GCTGGTGCAGAGAGACAC | U08188.1 |
|  | R: GAAAGCAGCGCGCACGCG |  |
| *FABP4* | F: AAGGTGAAGAGCATCATAACCCT | BC002148.1 |
|  | R: TCACGCCTTTCATAACACATTCC |  |
| *LPL* | F: TGAAGGACCTTATCGCATTGC | BC003305.1 |
|  | R: GCATGGGAAGCATTTTGTTGT |  |
| *CD36* | F: TGGAGCTGTTATTGGTGCAG | NM_001159555.2 |
|  | R: TGGGTTTTGCACATCAAAGA |  |
| *β-actin* | F: ACCACAGCCGAGAGAGAAAT | NM_007393.5 |
|  | R: GACCTGACCATCAGGGAGTT |  |

^1^Hamp, hepcidin antimicrobial peptide; FABP, fatty acid-binding protein; G3PDH, glycerol-3-phosphate dehydrogenase; SREBP, sterol regulatory element-binding transcription factor; PPAR*γ*, peroxisome proliferator-activated receptor-*γ*; ACC, [acetyl](https://cn.bing.com/dict/clientsearch?mkt=zh-CN&setLang=zh&form=BDVEHC&ClientVer=BDDTV3.5.1.4320&q=%E4%B9%99%E9%85%B0%E8%BE%85%E9%85%B6A%E7%BE%A7%E5%8C%96%E9%85%B6)-COA [carboxylase](https://cn.bing.com/dict/clientsearch?mkt=zh-CN&setLang=zh&form=BDVEHC&ClientVer=BDDTV3.5.1.4320&q=%E4%B9%99%E9%85%B0%E8%BE%85%E9%85%B6A%E7%BE%A7%E5%8C%96%E9%85%B6); FAS, fatty acid synthetase; ACLY, ATP-citrate lyase; SCD1, stearoyl-COA desaturase; CPT, carnitine palmitoyl acyl-CoA transferase; ApoB, apolipoprotein B; ACSL, long-chain acyl-CoA synthetase; ATGL, adipose triglyceride lipase; HSL, hormone-sensitive lipase; LPL, lipoprotein lipase; CD36, the cluster of differentiation 36.
